# A comprehensive dataset of board of directors attributes of Pakistan stock exchange listed non-financial firms

**DOI:** 10.1016/j.dib.2022.107879

**Published:** 2022-01-28

**Authors:** Sattar Khan, Yasir Kamal

**Affiliations:** a Scholar at Institute of Management Sciences Peshawar, Pakistan; b Associate Professor, at Institute of Management Sciences Peshawar, Pakistan

**Keywords:** Board of directors, Audit committee, Corporate governance, Panel data, Non-financial firms, Pakistan stock exchange

## Abstract

The data set of this article is related to the paper “Do Board of Directors’ Characteristics Matter in Restraining Earnings Manipulation? Empirical Evidence from Pakistan Stock Exchange” Khan and Kamal, (2021) [1]. This data article presents the raw data of 315 non-financial listed firms of the Pakistan Stock Exchange (hereafter, PSX). The data relating to the Board of Directors has been manually extracted from 1890 annual reports of the concerned companies and the financial data were extracted from State Bank of Pakistan sources, business-recorder^1^ and PSX Data portal^2^. The dataset is an unbalanced panel data consisting of 1882 firms’ year's observations from 2014 to 2019 based on 315 non-financial listed firms consists of 20 variables. This paper's dataset is valid and useful for researchers/scholars in many ways such as linking the board of directors to corporate governance, internal governance vs external governance, audit quality, financial reporting quality, firm performance, industry performance, corporate social responsibility and government regulation.

## Specifications Table

**Table:** 

Subject	Business, Management and Decision Science (Accounting & Finance)
Specific subject area	Corporate Governance & Board of Directors
Type of data	Panel Data Table in Excel & dta (Stata) File
How the data were acquired	The data relating to the board of directors has been hand-picked from the published annual reports of the concerned non-financial companies of PSX. In the annual reports, the director's data has been collected from directors’ reports to shareholders, directors’ profiles, statements of compliance and company information. Lastly, the financial data has been extracted from the State Bank of Pakistan (hereafter, SBP) published Balance Sheet Analysis, PSX Data portal and business recorder websites.
Data format	Raw and Analyzed Data
Description of data collection	Board of Directors’ attributes such as expertise, independence, female directorship, CEO Duality, CEO expertise, Audit Committee independence and Audit Committee Expertise data has been manually collected from 1890 annual reports of the 315 non-financial listed firms for the period 6 years ranging from 2014 to 2019.
Data source location	Pakistan, an emerging market economy. The data sources is annual reports of the Pakistan Stock Exchange 315 non-financial listed companies. In particular, in the annual reports the data is collected from directors’ reports to shareholders, directors’ profiles, statements of compliance and company information.
Data accessibility	The Dataset is attached with this article
Related research article	[1] S. Khan and Y. Kamal, “Do Board of Directors’ Characteristics Matter in Restraining Earnings Manipulation? Empirical Evidence from Pakistan Stock Exchange,” City Univ. Res. J., vol. 11, no. 1, pp. 60–83, 2021.

## Value of the Data

• This dataset is beneficial for many parties, because it may be considered comprehensive and complete data related to companies’ board structure and making it a valuable resource for Board of Directors (hereafter, BOD) attributes. This dataset may help related researchers to understand the dynamics of BOD in non-financial firms of PSX.
• Researchers/scholars of the Corporate Governance (hereafter, CG) & CG regulations, firms’ managers, investors, minority shareholders and regulator (s) may use this dataset for exploring board of directors’ related issues, drivers and challenges. Moreover, the regulator (SECP) may use it to check the compliance of CG codes in the Pakistani context.
• This dataset related to the board of directors may be used by CG researchers by linking it with firm performance, investment efficiency, firm diversification strategies, audit quality, accrual quality, risk aversion, managerial decision and different CG attributes.
• The dataset related to this article may be used to construct a BOD Index similar to [2] which may be linked with above-mentioned variables. Moreover, the Board Index and Board attributes may be checked with comparative analysis and by using different theoretical lenses.

## 1 Data Description

The data included in this article is an unbalanced panel that is related to the BOD attributes and firm characteristics. The dataset is consists of 1882 firms’ years’ observation of 315 non-financial listed firms for 27 sectors of PSX. The data collection period is from 2014 to 2019. Table 1 shows the sample selection procedure, which shows how the sample is reduced or comes up to 315 non-financial firms of PSX.

**Table 1:** Procedures of sample selection.

Total firms listed in the PSX in March 2021	**550**
**Less:** Financial firms	**(132)**
	**418**
**Less:** Annual Reports Missing, Defaulter and Delisted firms for the said period	**(93)**
	**325**
**Less:** Firms with missing Board of Directors & Audit Committee information	**(10)**
**Firms selected from the for the study**	**315**

In Table 2 the BOD attributes data is given along with description and data source, it is mainly collected from the annual reports of the companies. The annual reports which are freely available on different sources are downloaded from PSX Data portal, concerned companies’ websites and opendoors.pk^3^. In the annual reports, the director's data has been collected from directors’ reports to shareholders, directors’ profiles, statements of compliance and company information. The data has been extracted to MS excel file, the BOD of variables are presented in the data file from Column F to Column M. The variable BSIZE, BMEET, BIND, and BEXP are taken as it is, while BDIV, CEOD and CEOEXP are calculated during the extraction stage and reported is binary variables. In Table 3 description of the audit committee and financial variables is given which is included in the data file (Excel & dta). The audit committee variables, Firm Age and BIG4 Auditors data has been collected from the companies’ annual reports while the data of financial variables such as Return on Assets (ROA), Financial Leverage and Firm Size has been retrieved from the business recorder website and SBP sources. The variables of Audit Committee and Financial variables are calculated/adjusted before it were included in the dataset. The arrangement of audit committee variables and financial variables in the dataset file is from Column N to Column U.

**Table 2:** Description of board of directors variables.

S. No.	Variables	Type of Data	Variables Description	Data Source	Symbol in data file
1	Board Independence	Percentage (%)	The Number of Independent directors/ Size of the board	Annual Report	BIND
2	Board Expertise	Percentage (%)	Those directors who have accounting and finance-related education/experience in the company board are divided by size of the board	Annual Report	BEXP
3	Board Female directorship	Percentage (%)	The Number of Female directors/Size of the board	Annual Report	BDIV(Num)
4	Board Female directorship (Dummy)	Binary	1 is Taken if there is the presence of female director(s) in the board otherwise zero	Annual Report	BDIV (D)
5	Board Size	Number (count)	The number of directors in the board	Annual Report	BSIZE
6	Board meetings	Number (count)	The number of board meetings in a financial year	Annual Report	BMEET
7	CEO Duality	Binary	An indicator 1 is taken if the CEO and Chairman of the Board are the same persons otherwise zero.	Annual Report	CEOD
8	CEO Expertise	Binary	An indicator 1 is taken if the CEO has Accounting and Finance experience otherwise zero.	Annual Report	CEOEXP

**Table 3:** Description of audit committee and financial variables.

S. No.	Variables	Type of Data	Variables Description	Data Source	Symbol in data file
**Audit Committee Related Variables**					

1	Audit Committee Independence	Percentage (%)	Number of Independent directors in audit committee/ Size of the Audit Committee	Annual Report	ACIND
2	Audit Committee Expertise	Percentage (%)	Those directors who have accounting and finance-related education/experience by the size of the audit committee	Annual Report	ACEXP
3	Firm Age	Number (count)	Number of Years since Incorporation of the Firm	Annual Report	FAGE
4	BIG 4 Auditors	Binary	A firm audited by BIG4 audit firm will take 1 and others it will take 0	Annual Report	BIG4

**Financial Variables**					

5	Return on Assets	Ratio	Earnings Before Tax/Total Assets	Business-recorder and PSX Data portal	ROA
6	Financial Leverage	Ratio	Short Term & Long Term Debt/Total Assets	Business-recorder and PSX Data portal	LEVE
7	Firm Size	Numeric	Taking Log of Total Assets Value	Business-recorder and PSX Data portal	FSIZE

In addition to, BOD, Audit Committee and financial variables, in the dataset file Column A is for companies Symbol, Column B is for year variable, Column C represents the firm complete name, in Column D the PSX sectors code is given while Column E shows us the Company ID. Moreover, Table 4 shows the sector-wise distribution of the sample, which indicates that the largest observations are from the textile sector and the smallest distribution of the sample is from the woolen & leather sectors.

**Table 4:** Pakistan stock exchange sector wise distribution of sample of the study.

S. No.	PSX Sector Name	Years						Total	Row %
		2014	2015	2016	2017	2018	2019		
1	Automobiles Assembler	12	12	12	12	12	12	72	3.83
2	Automobiles Parts & Accessories	7	7	7	7	7	7	42	2.23
3	Cable & Electrical Goods	5	5	5	5	5	5	30	1.59
4	Cement	19	19	19	19	19	19	114	6.06
5	Chemical	27	27	27	27	27	27	162	8.61
6	Engineering	11	11	11	11	11	11	66	3.51
7	Fertilizer	5	5	5	5	5	5	30	1.59
8	Food & Personal Products	17	17	17	17	17	17	102	5.42
9	Glass & Ceramics	8	8	8	8	8	8	48	2.55
10	Leather & Tanneries	1	1	1	1	1	1	6	0.32
11	Miscellaneous	8	8	8	8	8	8	48	2.55
12	Oil & Gas Exploration Co	4	4	4	4	4	4	24	1.28
13	Oil & Gas Marketing Co	8	8	8	8	8	8	48	2.55
14	Paper & Board	7	7	7	7	7	7	42	2.23
15	Pharmaceuticals	9	9	9	9	9	9	54	2.87
16	Power Generation & Distribution	13	13	13	13	13	13	78	4.14
17	Refinery	5	5	5	5	5	5	30	1.59
18	Sugar & Allied Industries	29	29	29	29	29	29	174	9.25
19	Synthetic & Rayon	7	7	7	7	7	7	42	2.23
20	Technology & Communication	12	12	12	12	12	12	72	3.83
21	Textile Composite	55	55	55	55	55	55	330	17.53
22	Textile Spinning	30	32	32	32	32	32	190	10.10
23	Textile Weaving	5	5	5	5	5	5	30	1.59
24	Tobacco	2	2	2	2	2	2	12	0.64
25	Transport	3	3	3	3	3	4	19	1.01
26	Vanaspati & Allied Industries	2	2	2	2	2	1	11	0.58
27	Woolen	1	1	1	1	1	1	6	0.32
	Total	**312**	**314**	**314**	**314**	**314**	**314**	**1882**	

	**Column %**	16.57811	16.68438	16.68438	16.68438	16.68438	16.68438		**100.00**

## 2 Experimental Design, Materials and Methods

This article contains comprehensive data of BOD attributes and firm characteristics relating to non-financial firms of PSX. The data collection process start from the listed firms of PSX which was 550 on March 2021. Then those 550 companies were separated into financial and non-financial firms, financial firms were excluded due to the reason specified in the literature [1]. Then one by one every non-financial firm(s) was/were taken from the PSX listed firms and searched in PSX Data portal for annual reports downloading, if annual reports were not available there (PSX Data Portal), then each and every company official site are searched out for annual reports, in addition to this, the opendoors.pk has also been used for annual reports downloading.

After downloading the annual reports of the firms, the next step was to screen out and extract the information of BOD attributes from annual reports to excel file similar to the methods adopted in the following studies [3,4] the board related information in annual reports were found in company information, directors’ reports to shareholders, directors’ profiles and statements of compliance. Following the aforementioned method, and screening out 1890 annual reports, we have formed an unbalanced panel dataset of 315 non-financial firms in Excel consisting of BOD variables such as Board Size, Board Meetings, Board Independence, Board Female Directorship, Board Expert Directors, CEO Duality, CEO Expertise, audit committee independence and audit committee expertise for the 6 years data period ranging from 2014 to 2019.

After BOD attributes data collection the second stage was to collect the firm characteristics data from the different sources such as businessrecoder, PSX Data Portal and Balance Sheet Analysis issued by SBP. In the first step, data was downloaded in raw form, and after that, it was merged with Board attributes data in STATA 14 by using the merge command. In such a way we formed a comprehensive data set of board attributes with the financial characteristics of the non-financial companies of PSX. Therefore, this dataset may be used to link it to the various CG related topics depending upon the researcher/scholars interest and specific theoretical lenses and perspectives.

## Ethics Statement

No Ethical concerns are involved in the data collection, because the data has been collected from the annual reports of the companies. In the annual reports are freely available on the official website of Pakistan Stock Exchange https://dps.psx.com.pk/.

## CRediT authorship contribution statement

**Sattar Khan:** Conceptualization, Formal analysis, Data curation, Writing – original draft, Visualization. **Yasir Kamal:** Writing – review & editing, Supervision.

## Declaration of Competing Interest

The authors declare that they have no known competing financial interests or personal relationships which have, or could be perceived to have, influenced the work reported in this article.

## Appendix Supplementary Materials

Image, application 1

Image, application 2

## Footnotes

3 https://opendoors.pk/annual-reports-list/.
